# Assessing trachoma elimination progress in districts with persistent trachoma, Western Province, Zambia

**DOI:** 10.1093/inthealth/ihaf041

**Published:** 2025-04-26

**Authors:** Consity Mwale, Chileshe Mboni, Ngonda Saasa, Chummy S Sikasunge, Chisanga Chelu, Tina Chisenga, Lubasi Sundano, Namasiku Siyumbwa Kunda, Phyllis M Moonga, Kaluba Lombe, Tabonga Naluonde, Sarah Boyd, Ana Bakhtiari, Cristina Jimenez, Emma M Harding-Esch, Michael Dejene, Freddie Masaninga, Nathan N Bakyaita, Davison Kwendakwema, Anthony W Solomon, Kangwa I M Muma

**Affiliations:** Kitwe Teaching Eye Hospital, Administration Department, Kitwe 10101, Zambia; Lusaka Apex Medical University, Faculty of Medicine, Lusaka 10101, Zambia; Kitwe Teaching Eye Hospital, Administration Department, Kitwe 10101, Zambia; Departments of Disease Control and Paraclinical Studies, School of Veterinary Medicine, University of Zambia, P.O. Box 32379, Lusaka 10101, Zambia; Departments of Disease Control and Paraclinical Studies, School of Veterinary Medicine, University of Zambia, P.O. Box 32379, Lusaka 10101, Zambia; Mansa General Hospital, Administration Department, Mansa 10101, Zambia; Ministry of Health, Directorates of Public Health and Clinical Care, Lusaka 10101, Zambia; Ministry of Health, Directorates of Public Health and Clinical Care, Lusaka 10101, Zambia; Ministry of Health, Directorates of Public Health and Clinical Care, Lusaka 10101, Zambia; Ministry of Health, Directorates of Public Health and Clinical Care, Lusaka 10101, Zambia; Sightsavers, Lusaka 10101, Zambia; Lions Aid Zambia, Lusaka 10101, Zambia; International Trachoma Initiative, The Task Force for Global Health, 330 West Ponce de Leon Ave, Decatur, GA 30030, USA; International Trachoma Initiative, The Task Force for Global Health, 330 West Ponce de Leon Ave, Decatur, GA 30030, USA; Neglected Tropical Diseases Department, Sightsavers, Haywards Heath, RH16 3BW, UK; Clinical Research Department, London School of Hygiene & Tropical Medicine, London, WC1E 7HT, UK; Neglected Tropical Diseases Department, Sightsavers, Addis Ababa 495, 1110, Ethiopia; World Health Organization, Lusaka 10101, Zambia; World Health Organization, Lusaka 10101, Zambia; Beverly Eye and Dental Clinic, Ndola 10101, Zambia; Global Neglected Tropical Diseases Programme, World Health Organization, Geneva, 1211, Switzerland; University Teaching Hospitals-Eye Hospital, Clinical Care, Private Bag, RW 1X, 10101, Lusaka Zambia; School of Medicine, University of Zambia, P.O. Box 50110, Lusaka 10101, Zambia; School of Medicine, Levy Mwanawasa Medical University, P.O. Box, 33991, Lusaka 10101, Zambia

**Keywords:** impact survey, mass drug administration, persistence, SAFE strategy, trachoma, trichiasis

## Abstract

**Background:**

Trachoma is a public health problem in Zambia. We aimed to estimate the prevalence of trachomatous inflammation—follicular (TF) in 1–9-y-olds and of trachomatous trichiasis (TT) in ≥15-y-olds after the implementation of trachoma elimination interventions to determine if the trachoma elimination thresholds had been achieved: <5% for TF in 1–9-y-olds and <0.2% TT for ≥15-y-olds.

**Methods:**

Two rounds of impact prevalence surveys in two evaluation units (EUs) comprising four districts of Western Province were conducted; the first in 2018, the second in 2023. All individuals aged ≥1 year from 30 households of 24 clusters in each EU were examined for trachoma. Data were captured electronically.

**Results:**

In 2018, TF prevalence in 1–9-y-olds was 13.9% in Kalabo/Sikongo and 17.9% in Shang'ombo/Sioma. Following further interventions, TF prevalence among 1–9-y-olds in 2023 was 7.7% and 12.5%, respectively. TT prevalences in ≥15-y-olds were 0.10% and 0.79% in 2018, and 0.4% and 0.2% in 2023, respectively.

**Conclusions:**

These EUs did not attain trachoma elimination thresholds as a public health problem. They fulfilled the WHO definition for persistent trachoma. Therefore, they warrant further investigation, including collection of *Chlamydia trachomatis* infection data, to inform future programmatic decision-making. Further TT surgical services are also needed.

## Introduction

Globally, trachoma is the most common infectious cause of blindness.^1^ It is caused by certain serovars of *Chlamydia trachomatis*, an intracellular bacterium.^2^ In April 2024, trachoma remained a public health problem in 39 countries globally, with 103 million individuals living in areas where they were at risk of recurrent ocular *C. trachomatis* infection.^3^ As part of the evidence required to be validated by WHO as having eliminated trachoma as a public health problem, a country must demonstrate that the prevalence of trachomatous inflammation—follicular (TF, a clinical marker of ocular *C. trachomatis* infection) in 1–9-y-old children (TF1–9) is <5% in each formerly endemic district, and the prevalence of trachomatous trichiasis (TT, the late stage of the disease where eyelashes from the upper eyelid touch the eyeball) unknown to the health system in adults aged ≥15 y is <0.2% in each formerly endemic district.^4^

Africa continues to be both the continent most severely afflicted with trachoma and the home of the greatest control efforts. The WHO-recommended strategy for trachoma elimination is known as ‘SAFE’: Surgery for TT; Antibiotics to treat *C. trachomatis* infection, usually implemented as mass drug administration (MDA); Facial cleanliness and Environmental improvement to limit transmission. Surveys to determine the prevalence of trachoma, and interventions to eliminate trachoma, are usually implemented at the evaluation unit (EU) level. EUs are generally equivalent to districts, and for trachoma elimination purposes, WHO defines them as ‘the normal administrative unit for health care management, consisting of a population unit between 100 000–250 000 persons’.^4^ Baseline surveys are conducted to determine if interventions are needed (if TF1–9 is ≥5%), impact surveys are conducted 6–12 mo following the last planned MDA round to determine if the elimination threshold (TF1–9 <5%) has been reached, and surveillance surveys are performed at least 2 y after an impact survey to demonstrate TF1–9 <5% has been sustained in the absence of MDA.^5^

Since 2007, the Zambian Ministry of Health has effectively implemented the SAFE strategy approach to eliminate trachoma,^6^ resulting in a decrease in the number of districts affected by the disease from 56 to 15. In Western Province, baseline surveys were conducted in 2012, which resulted in six annual antibiotic MDA rounds (Table 1). For TT management, full geographic coverage^7^ has been conducted in Shang'ombo and Sioma. To inform the next steps, we conducted impact surveys in Kalabo, Shang'ombo, Sikongo and Sioma post-MDA to determine whether the TF and TT elimination prevalence thresholds had been met.

**Table 1.** Baseline trachomatous inflammation—follicular (TF) prevalence among 1–9-y-olds and estimated antibiotic mass drug administration (MDA) coverage (number of individuals treated/estimated population from most recent census), Western Province, Zambia

			Estimated coverage and year in which MDA was conducted						
District	Year of baseline survey	Baseline TF prevalence (%)	2015	2016	2017	2018		2021	2022
Kalabo	2012	42.1	**89%	96%	99%	Trachoma impact survey	73%	88%	84%
Sikongo				131%	83%		107%	106%	88%
Shang'ombo	2012	15.2	***136%	70%	93%		74%	101%	83%
Sioma				79%	73%		75%	94%	86%

** MDA coverage before Sikongo was split from Kalabo.

*** MDA coverage before Sioma was split from Shang'ombo.

## Materials and methods

### Study sites

Two impact survey rounds were conducted in the districts of Kalabo, Sikongo, Shang'ombo and Sioma, grouped as two EUs: the first round was carried out in 2018, and the second in 2023.

### Survey design

The surveys utilised a three-stage cross-sectional approach, which involved sampling wards within EUs, clusters within wards and households within clusters. Wards, which have well-defined geopolitical boundaries, population sizes and structures, were considered as the most basic administrative unit in the district.

### Population

The target populations of the surveyed districts were as shown in Table 2.

**Table 2.** Evaluation unit (EU) formation and population sizes for trachoma impact surveys conducted in Western Province, Zambia

Evaluation Unit (EU)	District	District population^a^	Total EU population^a^	Population of 1–9-y-olds	Number of wards	Number of clusters
Kalabo/Sikongo	Kalabo	32 643	75 191	10 772	8	24
	Sikongo	42 548		18 645	12	
Shang'ombo/Sioma	Shang'ombo	51 818	102 591	21 762	10	24
	Sioma	50 773		17 729	10	

a Source: Zambia Statistics Agency (2011).

### Sample size calculation

The required sample size was computed using the single population proportion for precision formula: n = DEFF × (z^2^ x p(1-p)/c^2^) x e, where DEFF (2.63) is the design effect; z (1.96) is the SD corresponding to 95% CIs; p (4%) is the expected prevalence; c (2%) is the absolute precision; and e (1.2) is an inflation factor to account for non-response. Computing these values gave a sample size (n) of 1164 children aged 1–9 y in each EU to be enumerated. The TT-only sample size was not calculated as this survey was powered to detect the TF prevalence in 1–9-y-olds.^8^

### Determination of the number of clusters in each EU

To calculate the required number of clusters for each EU, the total number of targeted children aged 1–9 y was divided by the average number of households that a team can comfortably survey per day (30) multiplied by the average number of 1–9-y-olds per household. The estimated mean number of 1–9-y-olds in Zambian families is 1.63. Consequently, a total of 24 clusters were required for each EU (1164/(30×1.63)).

### Sampling of wards and clusters

Selection of the clusters was performed using a two-stage sampling technique. First, wards were selected with probability proportional to population size (PPS) (i.e. the wards were chosen with a probability proportionate to their population size), using systematic sampling.^9^ Second, within selected wards, clusters were again selected using PPS. The allocation of clusters to each ward in a given district was equally based on PPS, that is, wards with larger population sizes were allocated more clusters (proportional to the population size of the ward). Third, the selection of households in each cluster was performed by utilising the compact segmentation method. The clusters were divided into segments by dividing the total number of households by 30. The lottery method was used to sample the final segment where 30 households were sampled.^8–10^

### Training of survey teams

Training of graders and recorders was conducted over 5 d, using the system described in the then-current Tropical Data Training Manual.^11,12^ Each survey team consisted of a grader, a data recorder, a driver and a local guide. For the 2018 surveys, graders were required to pass an inter-grader agreement (IGA) test with a kappa score of ≥0.7 for the diagnosis of TF against a reference grader; first on a set of 50 photographs, second by grading 50 children in the field. For the 2023 surveys, graders underwent a series of three classroom tests: (i) a follicle identification test; (ii) classroom photo-based IGA tests; and (iii) Classroom Objective Structured Clinical Examinations (OSCEs) of examination techniques. They then conducted a field-based OSCE. In addition, graders were trained on TT case identification. For the 2018 surveys, there was no assessment for TT grading; for the 2023 surveys, TT grading was assessed using an OSCE. IGAs to assess TT grading are not possible due to the rarity of TT cases. Recorders were trained to accurately record household and individual examination data in Android smartphones using the Tropical Data Open Data Kit application (The Task Force for Global Health Decatur, GA USA), and required to pass a recorder reliability test. The Tropical Data application is a customised version of Open Data Kit, accessible at no cost on the Google Play Store (https://play.google.com/store/apps/details?id=com.tropicaldata.collect.android). Successful graders and recorders then practised working together, before becoming survey team members. Teams were supervised in the field by an ophthalmologist, who spent a minimum of 1 d with each survey team per week to provide hands-on technical support.

### Data collection

Prior to participation, each participant was provided with comprehensive material that was read to them in their local language. Adult household members granted verbal consent on behalf of individuals aged <18 y, and for inspection of the latrines and handwashing facilities. Consent for examination was recorded on Android smartphones.

All data were electronically collected using the Tropical Data Open Data Kit application (https://tropicaldata.knowledgeowl.com/help). The data collected were stored on the smartphone's internal memory until internet connectivity was established, at which point they were uploaded to a Cloud-based server accessible 24/7 only by selected staff members of the Ministry of Health. Data were accessible via the password-protected Tropical Data website (https://www.tropicaldata.org/projects). The Ministry of Health possessed, verified and endorsed the collection and quality of data.

Household Global Positioning System coordinates were collected. Data were collected on access to drinking water sources and latrines.

All individuals aged ≥1 y, resident in a selected household at the time of the study, were eligible for eye examination. The grader looked for signs of active trachoma (TF and/or trachomatous inflammation—intense), trichiasis and trachomatous scarring in those with trichiasis.^13^ In the 2023 surveys, trichiasis was recorded separately for the upper and lower eyelids, and the grader counted the number of eyelashes touching the eyeball and the number of eyelashes epilated (recent epilation) from the affected eye, and reported the count to the recorder. The grader used 2.5× magnifying binocular loupes, and sunlight or a torch for illumination. In the 2023 surveys, follicle size guides were used to aid the diagnosis of TF.^14^

Individuals identified as having trichiasis were asked additional questions to explore their access to information and service for TT surgery, to determine whether the TT was known to the health system. The definition of a health worker in Zambia for the TT management questions was any person engaged in the promotion, protection or improvement of the health of the population. In the 2023 surveys, to confirm the response to the health management questions, the grader also looked for evidence of a surgical scar and had subsequent discussions with the participant to confirm TT management status.^8^

### Statistical analysis

The Ministry of Health approved data management support from Tropical Data. The data were analysed using the Tropical Data methodology as applied in trachoma prevalence surveys^8,15^ in R. Briefly, EU-level TF1–9 was calculated as the mean of cluster-level proportions adjusted for age in 1-y bands against the most recent census. The TT prevalence in those aged ≥15 y was calculated in a similar manner, but adjusted for gender and age in 5-y bands.

## Results

Of the 4383 children aged 1–9 y enumerated, 4297 (98%) were examined (Table 3). Of 6034 adults aged ≥15 y enumerated, 5654 (95%) were examined. The prevalence of TF1–9 in Shang'ombo/Sioma was 17.9% (95% CI 12.6 to 22.6%) in 2018 and 12.5% (95% CI 8.3 to 17.2%) in 2023. In Kalabo/Sikongo TF1–9 was 13.9% (95% CI 8.8 to 20.7%) in 2018 and 7.7% (95% CI 4.4 to 11.9%) in 2023.

**Table 3.** Post-intervention prevalence of trachomatous inflammation—follicular (TF) in 1–9-y-olds and trachomatous trichiasis (TT) unknown to the health system in ≥15-y-olds, and proportion of households with water, sanitation and hygiene access

District (year)	Number of house holds	Number of 1–9-y-olds enumerated	Number of 1–9-y-olds examined	Number of adults enumerated	Number of adults examined	TF prevalence (95% CI)	TT unknown to the health system prevalence (95% CI)	Proportion of households with an improved drinking water source within 30 min of the house (%)	Proportion of households with an improved latrine (%)
Shang'ombo/Sioma (2018)	742	1229	1200	1538	1440	17.9 (12.6 to 22.6)	0.8 (0.5 to 1.2)	25.9	0.4
Kalabo/Sikongo (2018)	750	1113	1098	1528	1463	13.9 (8.8 to 20.7)	0.1 (0.0 to 0.2)	34.4	1.7
Shang'ombo/Sioma (2023)	719	995	979	1488	1395	12.5 (8.3 to 17.2)	0.2 (0.1 to 0.5)	42.3	0.3
Kalabo/Sikongo (2023)	720	1046	1020	1480	1356	7.7 (4.4 to 11.9)	0.4 (0.1 to 0.9)	43.1	1.3

TT unknown to the health system for the evaluation unit surveyed in 2018 was defined as trichiasis of the upper and/or lower eyelid, whereas TT unknown to the health system in the 2023 surveys was defined as trichiasis of upper eyelid only.

The prevalence of TT unknown to the health system in those aged ≥15 y was 0.8% (95% CI 0.5 to 1.2%) in 2018 and 0.2% (95% CI 0.1 to 0.5%) in 2023 for Shang'ombo/Sioma; and 0.1 (95% CI 0.0 to 0.2%) and 0.4 (95% CI 0.1 to 0.9%) in 2018 and 2023, respectively, for Kalabo/Sikongo (Table 3).

In both EUs at both timepoints, <50% of households had access to drinking water within 30 min of the house, and only about 1% had access to a latrine (Table 3).

## Discussion

In these trachoma impact surveys of two EUs, covering four districts of Western Province, Zambia, the prevalence thresholds for trachoma elimination have not been met: TF1–9 remains ≥5% and TT unknown to the health system ≥0.2% (except for the 2018 estimate in Kalabo/Sikongo, where TT was 0.1%). This finding is despite six MDA rounds of azithromycin, with the most recent (2022) average MDA coverage in these districts of 85%. The decision to implement six MDA rounds was based on the finding that TF prevalence estimates were >10% at both the baseline surveys and the subsequent impact surveys, and with reference to relevant WHO recommendations (WHO recommends implementing three rounds of antibiotic MDA in areas where the prevalence of TF among 1–9-y-olds is 10% to 30%;^9^ and the minimum treatment coverage should be 80%^16^).

To address trachoma elimination challenges, the Western Provincial Health Office (WPHO) is employing the WHO-endorsed SAFE strategy using multisectoral interventions. This includes surgical intervention for TT with the WPHO conducting TT surgical outreach visits; further antibiotic MDA rounds with azithromycin, provided by Pfizer for elimination programmes via the International Trachoma Initiative; maintenance of facial cleanliness; and improvements to the environment, particularly in access to water and sanitation.^17,18^

In general, Zambia has made significant progress towards trachoma elimination, reducing the number of endemic districts from 56 to 15 from 2007 to 2023.^19^ The ongoing existence of trachoma as a public health problem in the Western Province is consistent with the experiences of several other countries that have employed the SAFE strategy but not always achieving desired outcomes.^20^ A WHO informal consultation held in December 2021 specifically addressed this issue of EUs failing to reach or sustain TF1–9 below the 5% elimination prevalence threshold. The following definitions were agreed: an EU with persistent active trachoma is one in which at least two impact surveys have TF1–9 ≥5% and have never had a survey with TF1–9 <5%; an EU with recrudescent active trachoma is one in which at least one surveillance survey estimates a TF1–9 ≥5%.^21^ Using this definition, Kalabo, Sikongo, Shang'ombo and Sioma districts have persistent active trachoma and may delay Zambia from joining the 21 countries that WHO had validated as having eliminated trachoma as a public health problem by November 2024.^22^

What underlies the phenomenon of persistent active trachoma? A variety of factors could contribute, including incomplete or incorrect implementation of the SAFE strategy, resistance of *C. trachomatis* to the antibiotics given as part of MDA and failure to control transmission through the F&E components of the SAFE strategy. Although the most recent MDA coverage in these districts ranged from 83% to 88%, inadequate antibiotic coverage could be one of the factors that led to persistent active trachoma in this setting. There is limited evidence for 80% being a biologically relevant threshold for effective coverage.^23^ An additional challenge for both maximising and estimating coverage is incomplete knowledge of the size and distribution of the population. This is an issue for every community-based public health programme. The latrines and handwashing facility findings from the 2018 and 2023 surveys in Western Province indicate some improvements in water access but continued challenges with sanitation. Although effective implementation of the F&E components to limit *C. trachomatis* transmission is also problematic, given the weakness of the evidence base for these interventions,^24,25^ it is important for the WPHO, local authorities and partners to continue lobbying and playing a leading role in the implementation of multisectoral water, sanitation and hygiene interventions.^26,27^

Relying solely on standard roll-out of antibiotic MDA and knowledge dissemination on facial cleanliness and environmental improvement is not always adequate to guarantee the long-term viability of active trachoma elimination.^28^ The persistence of active trachoma should be seen as a reminder to not rely on medical techniques alone, as the resources dedicated to preventing blindness are limited. To address the challenge, the WPHO is setting out to undertake evidence-based research to improve the SAFE strategy, particularly by enhancing surgical quality, optimising antibiotic MDA, knowledge, attitude and practice surveys, as well as multisectoral investment in water, sanitation and hygiene.^29^ A more thorough comprehension of the strategies for improving sanitation, hygiene and environmental conditions is undoubtedly a pressing requirement.^30^ For EUs with persistent active trachoma, global evidence-based recommendations are also needed on whether to continue with annual MDA, increase the frequency of MDA to more than once a year, or adopt the cautious approach of monitoring the situation before taking further action; the research planned in Zambia can contribute to this evidence base.

Given the declines in specificity of TF as a marker of conjunctival *C. trachomatis* infection after A, F and E interventions have commenced,^31,32^ ensuring that these interventions are indicated for trachoma elimination purposes is also important. For this reason, the next round of investigations in Zambia will be enhanced trachoma impact surveys, which include collection of dried blood spots and conjunctival swab samples for serology and *C. trachomatis* nucleic acid amplification testing (NAAT), respectively. Serology and NAAT are helpful in allowing inference about the true trachoma transmission intensity^33,34^ and are likely to be increasingly employed by national programmes.^21^

## Conclusion

These EUs did not attain trachoma elimination thresholds as a public health problem. They fulfilled the WHO’s definition for persistent trachoma. Therefore, they warrant further investigation, including collection of *C. trachomatis* infection data, to inform future programmatic decision-making. Further TT surgical services are also needed.

## Data Availability

The data utilised for this study and report may be obtained from the corresponding author. The data are owned by the Ministry of Health, Zambia.

## Disclaimer

The authors alone are responsible for the views expressed in this article and they do not necessarily represent the views, decisions, or policies of the institutions with which they are affiliated, USAID or the United States Government. The boundaries and names shown and the designations used on the maps in this publication do not imply the expression of any opinion whatsoever on the part of the authors, or the institutions with which they are affiliated, concerning the legal status of any country, territory, city or area or of its authorities, or concerning the delimitation of its frontiers or boundaries.

Copyright © 2024, © The Author(s) 2024. Published by Oxford University Press on behalf of Royal Society of Tropical Medicine and Hygiene.

This is an open access article distributed under the terms of the Creative Commons CC BY license, which permits unrestricted use, distribution, and reproduction in any medium, provided the original work is properly cited. You are not required to obtain permission to reuse this article.
